# Regulation of myeloid cells by activated T cells determines the efficacy of PD-1 blockade

**DOI:** 10.1080/2162402X.2016.1232222

**Published:** 2016-09-09

**Authors:** Nina Eissler, Yumeng Mao, David Brodin, Philippa Reuterswärd, Helene Andersson Svahn, John Inge Johnsen, Rolf Kiessling, Per Kogner

**Affiliations:** a Childhood Cancer Research Unit, Q6:05, Department of Women's and Children's Health, Karolinska Institutet, Stockholm, Sweden; b Cancer Center Karolinska, R8:01, Department of Oncology-Pathology, Karolinska Institutet, Stockholm, Sweden; c Bioinformatics and Expression Analysis Core Facility, Department of Biosciences and Nutrition, Novum, Karolinska Institutet, Huddinge, Sweden; d Division of Proteomics and Nanobiotechnology, Science for Life Laboratory, KTH Royal Institute of Technology, Stockholm, Sweden

**Keywords:** Colony stimulating factor 1 receptor (CSF-1R) inhibition, myeloid cell repolarization, neuroblastoma, PD-1 checkpoint blockade, purinergic enzymes

## Abstract

Removal of immuno-suppression has been reported to enhance antitumor immunity primed by checkpoint inhibitors. Although PD-1 blockade failed to control tumor growth in a transgenic murine neuroblastoma model, concurrent inhibition of colony stimulating factor 1 receptor (CSF-1R) by BLZ945 reprogrammed suppressive myeloid cells and significantly enhanced therapeutic effects. Microarray analysis of tumor tissues identified a significant increase of T-cell infiltration guided by myeloid cell-derived chemokines CXCL9, 10, and 11. Blocking the responsible chemokine receptor CXCR3 hampered T-cell infiltration and reduced antitumor efficacy of the combination therapy. Multivariate analysis of 59 immune-cell parameters in tumors and spleens detected the correlation between PD-L1-expressing myeloid cells and tumor burden. *In vitro*, anti-PD-1 antibody Nivolumab in combination with BLZ945 increased the activation of primary human T and NK cells. Importantly, we revealed a previously uncharacterized pathway, in which T cells secreted M-CSF upon PD-1 blockade, leading to enhanced suppressive capacity of monocytes by upregulation of PD-L1 and purinergic enzymes. In multiple datasets of neuroblastoma patients, gene expression of *CD73* correlated strongly with myeloid cell markers *CD163* and *CSF-1R* in neuroblastoma tumors, and associated with worse survival in high-risk patients. Altogether, our data reveal the dual role of activated T cells on myeloid cell functions and provide a rationale for the combination therapy of anti-PD-1 antibody with CSF-1R inhibitor.

## Abbreviations

A2A receptor: adenosine A2A receptor
ATP: adenosine triphosphate
CD: cluster of differentiation
cDNA: copyDNA
CFSE: carboxyfluorescein succinimidyl ester
COX-2: cyclooxygenase-2
CSF-1R: colony stimulating factor 1 receptor
CTLA-4: cytotoxic T-lymphocyte-associated protein 4
cvSE: coefficient scores and cross-validation standard errors
CXCL9: 10, and 11, chemokine (C-X-C motif) ligands 9, 10, and 11
CXCR3: C-X-C motif chemokine receptor 3
DC: dendritic cell
DNA: deoxyribonucleic acid
FACS: fluorescence activated cell sorting
IDO: indoleamine-2,3-dioxygenase
IFN: interferon
IL: interleukin
Lym-med: supernatants conditioned by activated lymphocytes
mAB: monoclonal antibody
M-CSF: macrophage colony-stimulation factor
MDSC: myeloid-derived suppressor cells
MLR: mixed lymphocyte reaction
NK cells: natural killer cells
OPLS-DA: orthogonal partial least squares discriminant analysis
PBMC: peripheral blood mononuclear cells
PBS: phosphate buffered saline
PCA: principle component analysis
PD-1: programmed cell death protein 1
PD-L1: programmed death-ligand 1
P2X7: P2X purinoceptor 7
RNA: ribonucleic acid
TAM: tumor-associated macrophage
TNF: tumor necrosis factor
VIP: variable importance scores

## Introduction

Immune checkpoint molecules are key rate-limiting pathways in controlling antigen presentation and T-cell activation.^1^ Blocking antibodies for this pathway, such as anti-CTLA-4 or anti-PD-1 mAb, lead to remarkable outcome in advanced stages of diseases and achieve response rates of up to 30% in metastatic melanoma patients that have failed standard treatments.^2,3^ However, solid tumors have the capacity to create a hostile environment that suppresses effector immune cell responses and supports growth and metastasis of tumor cells.^4^ In human cancers, the systemic and local accumulation of suppressive myeloid cells, such as myeloid-derived suppressor cells (MDSCs) or tumor-associated macrophages (TAMs) are documented to pose major challenges for the establishment of long-lasting antitumor immunity after these novel therapies.^5-7^

Several approaches targeting immuno-suppressive mechanisms are currently under evaluation in combination with checkpoint blockade antibodies.^8^ In tumor-bearing mice, concurrent blockade of cyclooxygenase-2 (COX-2), which is a key enzyme in inducing suppressive myeloid cells^9,10^, significantly enhanced the antitumor effects of checkpoint inhibitors.^11^ In addition, targeting immune-suppression mediated by indolamine-2,3-dioxygenase (IDO) demonstrated promising synergistic effects when combined with checkpoint blockade antibodies.^12-14^ This approach has recently shown efficacy in a phase I clinical trial together with anti-CTLA4 antibody Ipilimumab in patients with metastatic melanoma (NCT02073123).

We and others have previously defined the importance of myeloid cells as immune suppressors and novel therapeutic targets in the childhood neural cancer neuroblastoma.^15-19^ A highly selective inhibitor (BLZ945) targeting CSF-1R signaling potently modulated suppressive myeloid cells^20^ in transgenic mice developing spontaneous aggressive neuroblastoma (TH-*MYCN* model).^21,22^ Further, combining BLZ945 with checkpoint blockade antibodies against the PD-1/PD-L1 axis led to superior tumor control when compared to antibody treatment alone.^20^ Antibody treatment alone showed marginal effects on suppressive myeloid cells, which could help explain the ineffectiveness of checkpoint blockade as a single treatment. However, the key components resulting in the superior efficacy of the combination treatment remain elusive.

In this study, we show that combination of CSF-1R inhibitor BLZ945 with antibody blocking of PD-1-signaling leads to significant increase of IFNγ induced chemokines CXCL9, 10, and 11 in myeloid cells. Disrupting signaling of these chemokines hampers T-cell infiltration into tumors and thereby disables tumor control by the combination treatment. *In vitro*, combination of Nivolumab with BLZ945 increases percentages of CXCR3+ activated human T cells. Upon PD-1 blockade, activated T cells release high levels of M-CSF and increase myeloid cell-mediated immune-suppression, while addition of BLZ945 or of inhibitors targeting adenosine pathways overcomes suppressive mechanisms induced by M-CSF on monocytes. These results highlight a dual role of activated T cells on myeloid cells and provide a rationale for the use of combinational immunotherapy.

## Results

### Combination of BLZ945 and PD-1 blockade leads to effective tumor growth reduction by removing immune-suppression

**Figure 1.**
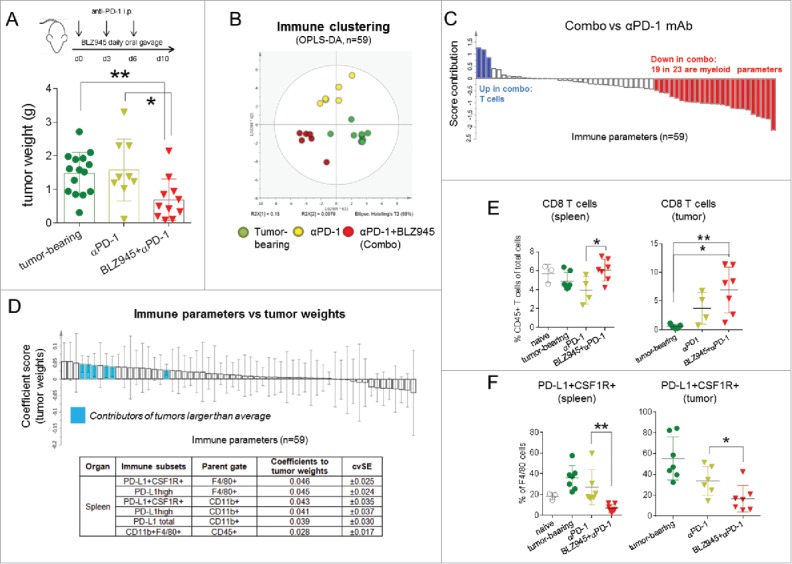
Expansion of T cells and reduction of suppressive myeloid cells results in antitumor activity. From the day spontaneous tumors were detected, TH-*MYCN* mice were treated by daily oral gavage of BLZ945 for 10 d combined with i.p. injections of anti-PD-1 antibody (12.5 mg/kg) on days 0, 3, and 6. Control mice were treated with anti-PD-1 antibody or left untreated. (A) Tumors were excised and tumor weights were compared among groups on day 10. Immune subsets and activation status of myeloid cells and lymphocytes were measured by flow cytometry in the spleens and tumors of control or treated mice (*n* = 59). (B) The clustering of the immune parameters was modeled and analyzed by multivariate analysis (OPLS-DA) using the SIMCA platform. (C) A direct comparison of the immune profile between combination treatment group and anti-PD-1 treated animals was demonstrated. (D) Next, tumor weights were set as Y variables and correlated with X variables (immune parameters, *n* = 59) using OPLS analysis. Immune parameters contributing to tumor burdens were highlighted in blue based on the coefficient scores and cvSE values. To validate the analysis, (E) frequencies of CD8 T cells and (F) PD-L1+CFS-1R+ macrophages were compared among different groups. *p < 0.05; **p < 0.01; non-parametric Mann–Whitney *U* test. Each dot in the scatter-dot plots represented an individual mouse. OPLS: orthogonal partial least squares analysis; OPLS-DA: orthogonal partial least squares discriminant analysis; and cvSE: cross-validation standard errors.

We treated transgenic TH-*MYCN* mice bearing detectable spontaneous neuroblastomas in the abdomen by oral gavage of BLZ945 in combination with i.p. injections of anti-PD-1 antibody for 10 d (Fig. 1A). Control mice remained untreated or were treated with anti-PD-1 antibody alone.

Although anti-PD-1 treatment as a single agent showed marginal therapeutic benefits, combination treatment of BLZ945 with anti-PD-1 antibody led to significant reduction of tumor growth when compared to untreated control mice (*p* < 0.004) or anti-PD-1 single treatment (*p* = 0.013) (Fig. 1A).

In order to dissect the relationships of 59 immune parameters (Table S4) assessed by flow cytometric analysis of spleens and tumor tissues in treatment and control groups, we performed multivariate analysis using the SIMCA platform. The 59 parameters analyzed defining the immune status in spleens and tumors of treated and control mice comprised frequencies of suppressive myeloid cells, i.e., macrophages (CD11b+F4-80+), monocytic (CD11b+Ly6c+Ly6glow) and granulocytic MDSCs (CD11b+Ly6c-Ly6g+), as well as T cell subsets, i.e., CD4^+^ and CD8^+^ T cells, and further, expression of activation and maturation markers on these immune subsets, as summarized in Table S4. The results showed high consistency in the principle component analysis (PCA) (Fig. S1A). To compare the immune profiles of different treatment groups, we utilized the OPLS-DA analysis and observed distinct clustering of the groups (Fig. 1B), demonstrating that overall immune cell frequencies and surface marker expressions are distinctly different among the control and treatment groups. Importantly, a direct comparison between the combination treatment (BLZ945+anti-PD-1) and anti-PD-1 single treatment group revealed clear enhancement of T-cell numbers in the combination group, while 19 out of 23 decreased immune parameters in this group were of myeloid lineage (Fig. 1C), confirming that the effects of CSF-1R inhibition were primarily exerted on myeloid cells.

To investigate which immune status contributed to the superior treatment outcome, we analyzed the relationship between immune parameters (*n* = 59, X variables) and tumor weights (Y variables) in the matching animals using the OPLS analysis by calculating coefficient scores and cvSE. Although none of the immune subsets were associated with smaller tumors, we identified several candidates that correlated with larger tumor burdens (Fig. 1D). In detail, the presence of splenic myeloid cells that expressed PD-L1 and CSF-1R at high levels was associated with inefficient antitumor immune responses. Next, we validated the results by comparing the frequencies of the immune cells and demonstrated that combination treatment significantly increased splenic T-cell numbers as well as T-cell infiltration into tumors (Figs. 1E and S1B), and reduced macrophages and MDSCs and their expression of PD-L1 and CSF-1R in spleens and tumors (Figs. 1F and S1C–F).

### Combination treatment increases expression of T-cell recruiting chemokines

**Figure 2.**
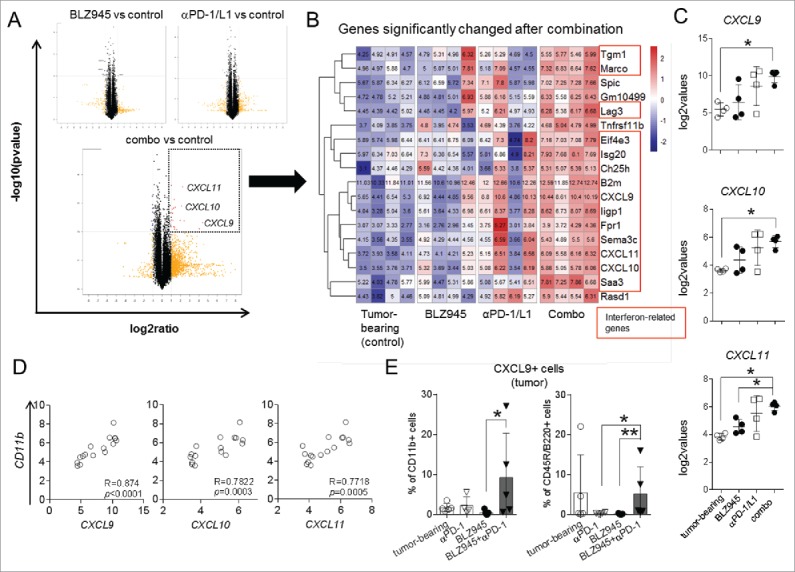
Combination therapy increases myeloid-cell-derived chemokines. Microarray analysis was conducted using mRNAs isolated from sized-matched tumors of control or treatment groups (four from every group). (A) Volcano plots identified genes that were significantly changed after treatments (dashed box) in comparison to untreated mice. (B) Heatmap of a list of significantly changed genes was demonstrated and interferon-regulated genes are marked in red boxes. (C) Expression of CXCL9, 10 and 11 was compared among groups and (D) correlated with myeloid cell marker *CD11b* calculated by Spearman test and two-tailed *t*-tests. (E) Intracellular expression of CXCL9 was confirmed in CD11b+ and CD45R/B220+ cells in the tumor tissues using flow cytometry. *p < 0.05; **p < 0.01; non-parametric Mann–Whitney *U* test. Microarray data accessible under GEO Series accession number GSE79485 (https://www.ncbi.nlm.nih.gov/geo/query/acc.cgi?acc=GSE79485).

To understand how CSF-1R inhibitor BLZ945 converted insufficient immune response induced by checkpoint blockade antibodies into a highly effective antitumor response, we performed microarray analysis of four individual tumors with comparable weights treated with either BLZ945, anti-PD-1/L1 mAbs, or the combination thereof and compared to untreated tumors (GEO Series accession number GSE79485; https://www.ncbi.nlm.nih.gov/geo/query/acc.cgi?acc=GSE79485). To our surprise, single treatments did not induce consistent changes in gene expression in comparison to control tumors. In contrast, the combination treatment induced significant upregulation of a number of genes (Fig. 2A), most of which were related to interferon pathways^23^ (Fig. 2B, highlighted in red). In particular, CXCL9, CXCL10, and CXCL11, which are interferon-induced chemokines, were prominently upregulated (Fig. 2C). In order to investigate the source of the chemokines, we correlated these genes with a number of immune and tumor markers. Of note, CXCL9, 10, and 11 expression correlated strongly with myeloid marker CD11b (*R* = 0.874, 0.7822, and 0.7718; Fig. 2D), as well as dendritic cell marker *CD11c* (*R* = 0.901, 0.945, and 0.903; Fig. S2A), but not with T or B cell markers, or the GD2 synthase *B4galnt1* tumor marker (Fig. S2B), indicating that myeloid cells are the major producers of these chemokines in the tumors.

To verify these results at the protein level, we conducted intracellular flow cytometry stainings of tumor materials. Our results demonstrated that the production of CXCL9 was significantly elevated in CD11b+ myeloid cells as well as CD45R/B220+ cells, a marker expressed on B cells, activated T cells, and several antigen presenting cells, in tumors of animals that had received the combination therapy (BLZ945+anti-PD-1), whereas single treatments resulted in low to undetectable levels of CXCL9 in these cells (Fig. 2E). These results underline the importance of interferon-regulated myeloid cell repolarization in priming potent antitumor immune responses after the combination treatment.

### Blockade of chemokine signaling abrogates efficacy of combination treatment

**Figure 3.**
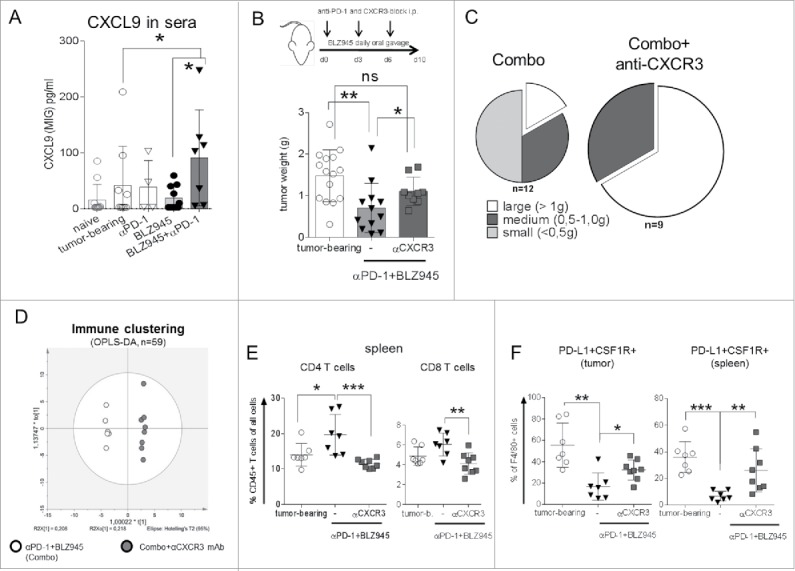
Blocking CXCR3 receptor abrogates *in vivo* treatment efficacy. Protein levels of CXCL9 in sera of treated mice were detected by Legendplex cytokine assays (A). To evaluate the functional roles of the CXCR3-CXCL axis, TH-*MYCN* mice were treated from the day spontaneous tumor growth was first palpable with BLZ945 (daily oral gavage) + anti-PD-1 antibody (12.5 mg/kg i.p. on days 0, 3, and 6) with or without concurrent blockade of CXCR3 by anti-CXCR3 antibody (12.5 mg/kg i.p., days 0, 3, and 6). (B) Tumor weights were measured on day 10 and (C) tumor weight distributions were summarized in pie charts. Immune parameters (*n* = 59) were monitored by flow cytometry in the combination group with or without CXCR3 blockade. (D) Clustering pattern of the immune parameters was analyzed by OPLS-DA analysis using the SIMCA platform. Frequencies of splenic and intra-tumoral (E) T cells or (F) PD-L1+CSF1R+ macrophages were compared. *p < 0.05; **p < 0.01; ***p < 0.001; non-parametric Mann–Whitney *U* test. Each dot in the scatter-dot plots represented an individual mouse.

Chemokines CXCL9, 10, and 11 signal via their common receptor CXCR3 and thereby recruit lymphocytes to inflammation sites and enable their penetration into the tumor tissues.^24,25^ To understand whether chemokine secretion is limited to the tumor tissue or plays a systemic role, we analyzed chemokine levels in sera of treated mice. Similar to tumor tissues, CXCL9 protein levels were substantially increased in sera of mice in the combination group (Fig. 3A), although no CXCL10 could be detected.

To assess the functional role of chemokine signaling via CXCR3, we administered an anti-CXCR3 antibody in addition to the combination treatment (Fig. 3B). Indeed, tumor growth reduction achieved by combination treatment was significantly hampered by CXCR3 blockade (*p* < 0.05, Fig. 3B) and tumor weight distributions clearly visualized the high numbers of large tumors (>1.0 g) and complete absence of small tumors (<0.5 g) (Fig. 3C). Blockade of CXCR3 resulted in very distinct overall immune clustering compared to the combination group when all 59 parameters (Table S4) were taken into consideration in the OPLS-DA analysis (Fig. 3D). In detail, CXCR3 blockade showed a trend to diminished T-cell infiltration into the tumors when compared to combination treatment, as well as significantly decreased T-cell frequencies in spleens (Figs. 3E and S2C) but led to the strong increase of immuno-suppressive myeloid cells that expressed PD-L1 and CSF-1R (CD115) at high levels (Fig. 3F).

### Blocking CSF-1R signaling on monocytes augments activation of lymphocytes induced by Nivolumab

In order to evaluate the effects of CSF-1R inhibition and PD-1 blockade on human immune cells, we utilized an *in vitro* model, where freshly isolated primary human immune cells were activated by mixed lymphocyte reactions (MLR) in CFSE-based proliferation assays.

Given the essential role of CXCR3 in T-cell infiltration in response to the combination immunotherapy *in vivo*, we measured the expression of CXCR3 on human T cells. Indeed, proliferating T cells expressed high levels of CXCR3 when activated with microbeads coated with anti-CD3/CD28 agonistic antibodies (Fig. S3A). Thus, we utilized the CFSE^dim^CXCR3+ T-cell populations as readouts for the subsequent functional analyses.

**Figure 4.**
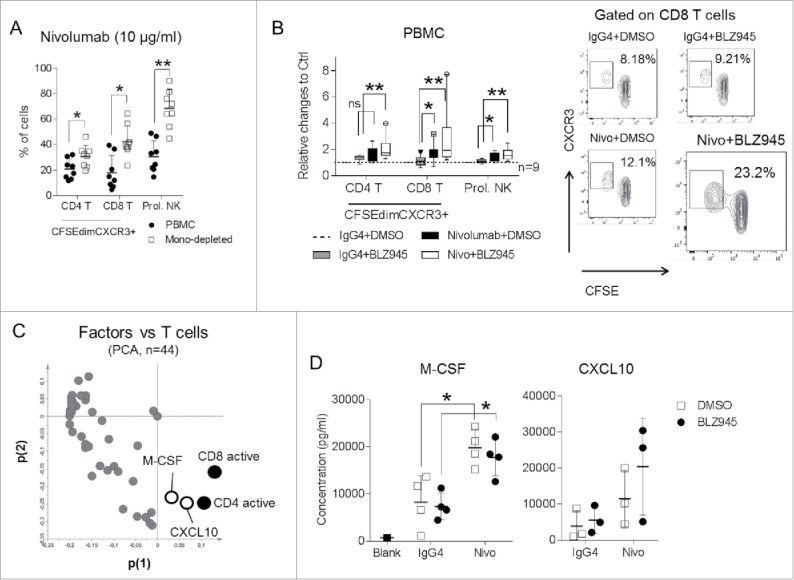
BLZ945 augments effects of Nivolumab on primary human lymphocytes. Peripheral blood mononuclear cells (PBMC) from healthy donors were labeled with CFSE and activated in mixed lymphocyte reactions (MLR) with Nivolumab (10 μg/mL) for 6 d and (A) frequencies of CFSE^dim^CXCR3+ T cells or CFSE^dim^ NK cells were compared when monocytes were present or absent. Alternatively, (B) PBMC were activated in the presence of a fully human IgG4 isotype control (10 μg/mL), DMSO, Nivolumab or combination of Nivolumab and BLZ945 (300 nM) and activation of lymphocytes were compared in nine independent donors in relation to the control group (IgG4 + DMSO). (C) Supernatants from three independent proliferation assays described in (B) were harvested and in total concentrations of 44 soluble factors were measured and visualized in relation to the T-cell activations in the loading plot of a PCA analysis. (D) Concentrations of M-CSF (*n* = 4) or CXCL10 (*n* = 3) were measured and demonstrated when PBMC were activated with IgG4 or Nivolumab. *p < 0.05; **p < 0.01; non-parametric Mann–Whitney *U* test. Each dot in the scatter-dot plots represented an individual blood donor. PCA: principle component analysis.

We hypothesized that monocytes hamper lymphocyte activation after PD-1 blockade. Indeed, depletion of monocytes prior to the functional assays (Fig. S3B) significantly increased the activation of lymphocytes induced by Nivolumab, which is demonstrated by enhanced proliferation of CXCR3+ CD4 and CD8 T cells as well as NK cells (Fig. 4A). Importantly, similar effects were observed when BLZ945 was added to PBMC in addition to Nivolumab, meaning that blockade of CSF-1R on monocytes is able to improve Nivolumab induced T-cell activation (Fig. 4B). Since CSF-1R is expressed at much higher levels on CD11b+ human myeloid cells (Figs. S3C and D), the additive effects of BLZ945 disappeared when monocytes were depleted (Fig. S3E).

Next, we measured 44 soluble factors in the culture supernatants harvested from three independent proliferation assays using human PBMC and analyzed the association of soluble factors with T-cell activation. As shown in the loading plot of the PCA analysis (Fig. 4C), M-CSF and CXCL10 demonstrated close associations with T-cell activation in our culture system and presented at substantially higher levels when anti-PD-1 antibody was added (Fig. 4D). To confirm that M-CSF was released from activated T cells, we measured intracellular expression of M-CSF when PBMC were activated with increasing doses of anti-CD3/CD28 agonistic antibodies and demonstrated that M-CSF was only upregulated on proliferating Ki67+ T cells (Fig. S4A). When T cells were activated with various numbers of allogeneic monocytes, we also observed higher levels of soluble M-CSF when more CFSE^dim^CXCR3+ T cells were generated (Fig. S4B).

### T-cell derived M-CSF enhances suppressive functions on healthy human monocytes

To investigate whether suppressive functions of primary human monocytes were modulated by restraining CSF-1R signaling, we tested a number of pharmacological inhibitors and blocking or neutralizing antibodies targeting suppressive mechanisms exploited by myeloid cells in combination with anti-PD-1 antibody in proliferation assays using human PBMC.

**Figure 5.**
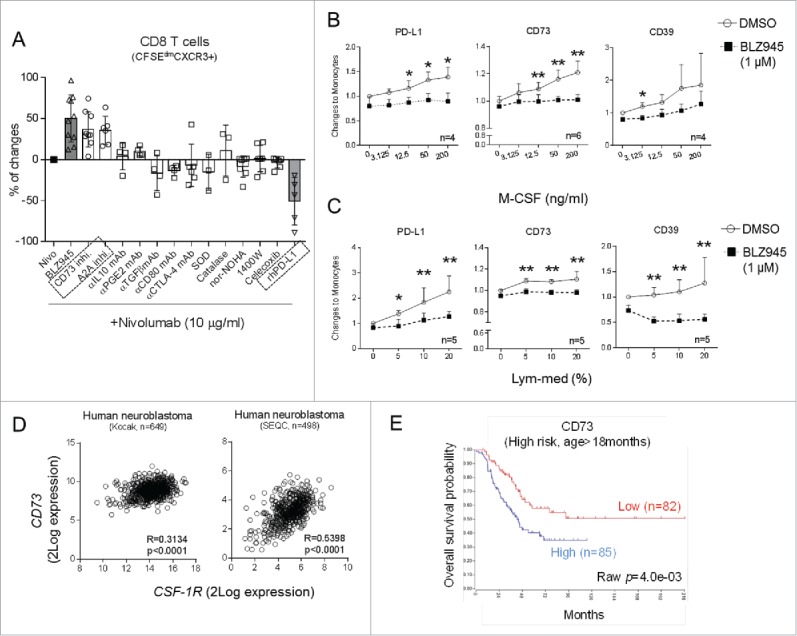
Monocyte-derived adenosine is enhanced by M-CSF produced by activated T cells. (A) Freshly isolated human PBMC were activated in mixed lymphocyte reactions (MLR) in the presence of Nivolumab (10 μg/mL) in combination with BLZ945 (gray bar, 300 nM) or antibodies or pharmacological compounds inhibiting various suppressive mechanisms and relative changes of CD8 T-cell activation to Nivolumab alone were summarized. Freshly isolated primary human monocytes were cultured in increasing concentrations of (B) recombinant human M-CSF or (C) media conditioned by activated lymphocytes. Expression of CD73, PD-L1, and CD39 was compared between monocytes treated with DMSO or BLZ945 (1 μM). (D) Correlations between gene expression of *CD73* and *CSF-1R* in human neuroblastoma tumor tissues were analyzed in two public patient datasets using Pearson correlation coefficients and 2-tailed T tests. (E) The prognostic value of *CD73* gene was demonstrated in high-risk patients who were older than 18 months. *p < 0.05; non-parametric Mann–Whitney *U* test.

Notably, attenuating enzymatic functions of CD73, as well as blocking adenosine A2A receptor, but not blocking other inhibitory mechanisms, improved activation of CD8 T cells (Fig. 5A). On the other hand, addition of recombinant human PD-L1 (Fig. 5A) or blocking co-stimulatory pathways reduced activation of T cells (Fig. S5A). To validate our findings, we treated isolated human monocytes with supernatants conditioned by activated lymphocytes (Lym-med) or recombinant human M-CSF. Both treatments induced rapid maturation of primary human monocytes and upregulated their surface expression of CD206 (Fig. S5B). Notably, expression of surface PD-L1 and key enzymes for adenosine production, CD73 and CD39, was increased on monocytes by Lym-med factors or human M-CSF. These effects were sufficiently abrogated by addition of CSF-1R inhibitor (Fig. 5B and C). Meanwhile, expression of HLA-DR, CD80, CD137L, and DC-SIGN or intracellular levels of M-CSF, IFNγ, and IDO were not altered by the treatments on healthy monocytes (unpublished observations). Although CD73 and CD39 were upregulated on subsets of lymphocytes, expression of both proteins was markedly higher on myeloid cells when PBMC were activated (Fig. S5C). Inhibiting adenosine pathway in addition to the anti-PD-1/BLZ945 combination did not further improve the activation of CD8 T cells, indicating the direct connection between CSF-1R signaling and adenosine production on monocytes (Fig. S5D).

In order to evaluate the clinical importance of myeloid cell-derived adenosine production, we analyzed six publicly available neuroblastoma patient datasets (in total 1687 entries) and demonstrated that expression of *CD73* and *CD39* genes was only consistently correlated with myeloid cell markers *CD163* or *CSF-1R* (Table 1 and Fig. 5D). A similar trend was also observed in tumor tissues harvested from TH-*MYCN* mice undergone various treatments (Fig. S6A). *M-CSF* expression in these tissues also strongly correlated with *CD73, CD39*, and *PD-L1*, indicating the involvement of M-CSF in the induction of myeloid cell suppressive mechanisms (Fig. S6B). Of note, expression of high levels of *CD73*, but not *CD39* (unpublished observations) in neuroblastoma tumors, was associated with worse overall survival in high-risk neuroblastoma patients (age > 18 mo) (Fig. 5E), whereas it had no influence on survival of low-risk patients (Fig. S6C).

**Table 1.** Correlation analysis of *CD39/CD73* in human neuroblastoma tumor samples.

		Correlation coefficent (*R*)					
Cell types	Patient datasets	Kocak (*n* = 649)	SEQC (*n* = 498)	Asgharzadeh (*n* = 249)	Seeger (*n* = 102)	Maris (*n* = 101)	Versteeg (*n* = 88)
Myeloid cells	CD73 vs CD163	0.326	0.467	0.602	0.332	0.529	0.466
	CD39 vs CD163	0.582	0.484	0.734	0.558	0.362	0.609
	CD73 vs CSF1R	0.314	0.540	NA	0.402	0.226	0.487
	CD39 vs CSF1R	0.530	0.496	NA	0.628	0.071	0.597
Tregs	CD73 vs FOXP3	−0.245	0.200	0.084	0.038	NA	0.020
	CD39 vs FOXP3	−0.239	0.108	0.035	0.019	NA	−0.008
B cells	CD73 vs CD19	0.107	0.239	0.301	0.207	0.060	0.245
	CD39 vs CD19	0.068	0.213	0.298	0.353	0.254	0.152
Tumors^*^	CD73 vs B4GALNT1	−0.050	−0.053	−0.069	−0.126	−0.112	−0.250
	CD39 vs B4GALNT1	−0.090	−0.114	−0.145	−0.040	−0.141	−0.126
	References	^51^	GEO ID: gse62564	TARGET data matrix	^52^	GEO ID: gse3960	^53^

* B4GALNT1 (β-1,4-N-acetyl-galactosaminyl transferase 1) is responsible for GD2 synthesis, which is a disialoganglioside commonly over-expressed in human neuroblastoma.

*R* > 0.4; Black: 0.2 < * R* < 0.4. NA: gene not available.

Despite the relevance of the adenosine pathway in neuroblastoma, treatment of TH-*MYCN* mice with a combination of anti-PD-1 and anti-CD73 antibodies did not lead to tumor growth inhibition, and a triple combination with CSF-1R inhibitor BLZ945 had to be discontinued due to severe weight loss in mice.

## Discussion

**Figure 6.**
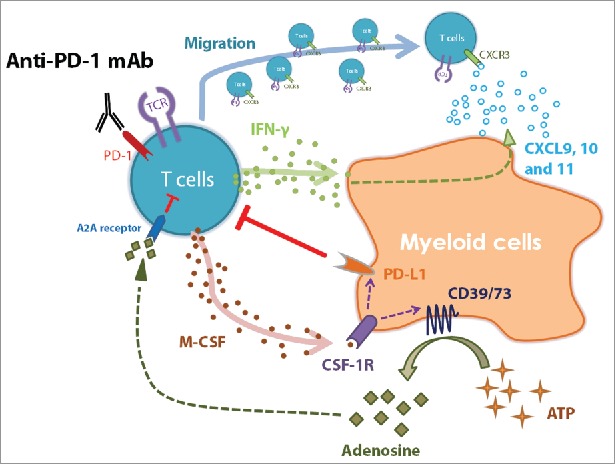
Regulation of myeloid cells by activated T cells in response to anti-PD-1 therapy. The graph summarizes the major findings of this study. Anti-PD-1 antibody treatment results in the release of IFNγ by activated T cells and re-activates myeloid cells to produce chemokines CXCL9, 10, and 11, which recruit T cells via CXCR3 signaling. At the same time, activated T cells secret M-CSF and enhance suppressive potentials of myeloid cells, such as upregulation of PD-L1 as well as CD39/CD73 expression. The adenosine catabolizing enzymes CD39/CD73 convert ATP to adenosine, which can inhibit T cells via A2A receptors.

Stimulated by the finding that CSF-1R antagonist enabled anti-PD-1 antibody to control tumor growth in the transgenic neuroblastoma mouse model (TH-*MYCN*), we aimed to dissect the key mechanistic events underlying this combination treatment. Our results revealed that the combination substantially reduced numbers of PD-L1-expressing suppressive myeloid cells *in vivo* and repolarized intra-tumoral myeloid cells to produce T-cell-recruiting chemokines. Exploiting *in vitro* models of human primary immune cells, we demonstrated the previously uncharacterized aspect that anti-PD-1 antibody enhanced the release of M-CSF from activated T cells and subsequently promoted suppressive functions on monocytes, mainly through elevating adenosine production and PD-L1 expression. In neuroblastoma patients, the enzymatic machinery of adenosine production was predominantly expressed on myeloid cells and correlated with poor survival in high-risk patients. The main findings are illustrated in Fig. 6.

Despite the clinical success of checkpoint blockade antibodies, a large number of patients failed to establish long-lasting antitumor immunity from the single agent treatment.^26^ This could be explained by various mechanisms exerted by solid tumors to grow, metastasize, and prevent antitumor immunity.^4^ The tumor microenvironment holds out several challenges against destruction by immune effector cells such as hypoxic environment and accumulation of suppressive cell types, including regulatory T cells and suppressive cells of the myeloid lineage.^27^

Suppressive myeloid cells have been demonstrated to play a key role in the development of several malignancies and have been correlated to increased stage of malignancies.^27-30^ In neuroblastoma, infiltration of TAMs was correlated to metastatic disease and worse prognosis.^15^ Targeting suppressive myeloid cells through CSF-1R inhibition provides a promising approach for solid cancers,^20,31,32^ particularly when combined with checkpoint inhibitors.^20,33^ Several clinical programs are currently evaluating the safety and therapeutic values of this combinatorial approach (NCT02452424, NCT02526017, NCT02323191, and starting in October 2016 NCT02829723). Therefore, it is of substantial interest to investigate the detailed mechanisms contributing to the superior effects of the combination treatment. Here, we demonstrated that anti-PD-1 antibody as a single treatment failed to control tumor growth, whereas the combination treatment with CSF-1R inhibitor BLZ945 showed superior tumor reduction.

Previous studies demonstrated the ability of single agent BLZ945 to repolarize myeloid cells;^31^ others showed that PD-1 blockade was able to induce IFNγ-related chemokine production.^34^ We found that only the combination of the two therapies was effective in achieving efficient immune-cell activation resulting in strong antitumor efficacy in the transgenic neuroblastoma mouse model.^20^ Similar to the observations made by Pyonteck et al.,^31^ BLZ945 single treatment did not abolish myeloid cells in the tumor, probably due to the presence of cytokines sustaining myeloid cell survival in the tumor microenvironment.^20^ Our further analysis revealed that in addition to the removal of suppressive myeloid cells, repolarization of myeloid cells by enhancing CXCL9, 10, and 11 production presented an essential regulator. Indeed, blocking their common receptor CXCR3 *in vivo* prevented the therapeutic effects, possibly due to counteracting trafficking of effector T cells across tumor vessels.^25^ These findings coincide with the recent notion that IFN-regulated chemokines CXCL9, 10, and 11 are of favorable clinical outcomes in patients with cancers.^35-38^ Hence, it is reasonable to hypothesize that chemokines derived from activated myeloid cells may contribute to a beneficial immune texture in the tumor microenvironment.

One of the key challenges of the current checkpoint blockade therapy is the early identification of patients who are likely to respond to the treatment. We were among the first to report that frequencies and phenotypes of myeloid cells were rapidly altered in response to anti-CTLA-4 blocking antibody Ipilimumab in melanoma patients and could be correlated to poor treatment outcome.^39-41^ However, potential predictive biomarkers remain to be discovered in patients receiving anti-PD-1 therapy. Previous reports proposed that expression levels of PD-L1 on tumor cells were associated with clinical outcomes of the treatment.^41^ In the murine model of this study, we monitored simultaneously 59 immune cell parameters and demonstrated that splenic myeloid cells expressing PD-L1 and CSF-1R at high levels were associated with larger tumor burdens. Given that PD-L1 is also expressed by various immune cells in the tumor tissues,^42^ these markers may also have predictive value in human cancers.

It is well described that suppressive myeloid cells inhibit effector T-cell functions.^5^ However, less is known about how activated T cells could influence the functions of suppressive myeloid cells. Pinton et al. recently described that IL-10 released by activated T cells sustained activities of murine MDSCs.^43^ We screened 44 soluble factors released by primary human immune cells after PD-1 blockade and identified a strong association between M-CSF production and T-cell activation. Similar to the *in vivo* efficacy, blocking CSF-1R signaling on primary human monocytes enhanced the effects of anti-PD-1 antibody. M-CSF is a crucial factor involved in myeloid cell homeostasis and described to be released by activated T cells during autoimmune conditions.^44,45^ Thus, PD-1 blockade is likely to trigger intrinsic negative feedback mechanisms, i.e., release of M-CSF as shown here, which attenuate the antitumor efficacy.

Fast-proliferating cells are featured by over-production of ATP, which could directly enhance suppressive functions of myeloid cells through P2X7 receptor.^46^ Alternatively, the enzymatic machinery led by CD39 and CD73 enables conversion of ATP to extracellular adenosine and suppresses T cells through various adenosine receptors.^47^ Therapeutic inhibition of CD73^48^ or adenosine A2A receptor^49,50^ improved antitumor efficacy of checkpoint inhibitors in preclinical models. We demonstrate in this study that T-cell-released factors or recombinant M-CSF promote monocyte differentiation and upregulate a number of suppressive mechanisms such as PD-L1 expression as well as adenosine pathways. Blocking CSF-1R signaling restrained the induction of these molecules and showed comparable effects to adenosine antagonists in restoring T-cell activation. Of note, expression of the *CD73* gene was strongly correlated with *CSF-1R* gene expression and predicted poor clinical outcome in patients with high-risk neuroblastoma. These findings provide deeper understanding of how blockade of CSF-1R in cancer patients might counterbalance various suppressive mechanisms exerted by myeloid cells. The strong correlation of *M-CSF* in tumors of TH-*MYCN* mice to T cell markers, as well as to suppressive markers, underlines the relevance of these findings in the *in vivo* situation.

In conclusion, we believe that T-cell activation in response to PD-1 blockade may play a dual role in regulating myeloid cells. On the one hand, myeloid cell repolarization that is driven by IFNγ guides T-cell infiltration by chemokines, whereas on the other hand, M-CSF or other inflammatory mediators released from T cells induce intrinsic resistant mechanisms through enhancing suppressive functions on myeloid cells. The described effects of anti-PD-1 antibody on T-cell activation, in particular M-CSF release, and consequently on myeloid cell suppressive functions provide a rationale for the combination therapy with CSF-1R inhibitor. Hence, we suggest that combinational strategies targeting multiple regulatory mechanisms in the tumor microenvironment are of particular advantage in designing novel antitumor immunotherapies.

## Materials and methods

### Animals

TH-*MYCN* mice^21^ were obtained from the Mouse Model of Human Cancer Consortium Repository (N16 backcross to the 129×1/SvJ background) and kept as continuous inbreeding. All animal studies were approved by the local authorities and conducted under the ethical permit number N42/14. For therapy studies, heterozygous mice were palpated every second day and treatment started on the day tumors were first palpable (day 0). For *in vivo* treatment, anti-PD-1 blocking antibody (clone RMPI-1.14, Bioxcell) was injected intraperitoneally (i.p.) on days 0, 3, and 6. Alternatively, a highly selective CSF-1R tyrosine kinase inhibitor BLZ945 (Novartis) was dissolved in 20% Captisol® at 16 mg/mL and delivered daily by oral gavage at the dose of 200 mg/kg^20,31^ in combination with the PD-1 blocking antibody. In some experiments, a CXCR3 blocking antibody (clone CXCR3-173, Bioxcell) was injected i.p. in addition to the combination. All antibodies were applied at a dose of 12.5 mg/kg. Spleens, tumors, and sera of treated or control mice were harvested on day 10. One part of the tumors was snap frozen in liquid nitrogen and saved at −80°C until further analysis. The other part of the tumors as well as spleens was homogenized, erythrocytes were lysed (BD PharmLyse™ buffer, BD Bioscience), and samples were analyzed by flow cytometry. Sera were separated from blood cells and frozen at −80°C until further analysis.

### Microarray analysis

RNA isolation and purification, as well as microarray analysis of snap frozen murine tumor tissue, were performed by the Bioinformatics and Expression analysis core facility (BEA) at Karolinska Institutet. RNA was isolated using QIAGEN RNeasy Mini Kit. Samples were homogenized using a QIAGEN TissueLyzer and further processed in a QIAGEN QIAcube.

75 ng of total RNA was reverse transcribed and labeled using the Affymetrix Whole Transcript (WT Plus) Assay. The resulting cDNA was hybridized to a GeneTitan plate (Mouse Gene 2.1 ST). Raw expression data were analyzed using Affymetrix Expression Console v1.4.1 using the RMA procedure. Sample groups were compared using unpaired *t*-tests.

### Isolation of human primary immune cells

Primary human peripheral blood mononuclear cells (PBMC) were isolated from buffy coats of healthy blood donors by Ficoll gradient centrifugation (GE Healthcare). In order to deplete monocytes, 10 μL RosetteSep human monocyte depletion cocktail (StemCell Technologies) was added to 10 mL buffy coat and incubated at room temperature for 20 min, prior to centrifugation. In order to purify primary human monocytes, PBMC were incubated with microbeads coated with anti-CD14 mAb followed by isolation using LS columns (Miltenyi Biotech).

### Functional analysis of human immune cells

Unless otherwise stated, all *in vitro* experiments were performed using IMDM medium (Invitrogen) supplemented with 10% pooled human AB serum (Karolinska University Hospital). To evaluate the effects of treatments, PBMC or monocyte-depleted PBMC (mono-dep cells) were incubated with 1.4 μM CellTrace™ CFSE (Life Technologies) for 6 min at room temperature. Cells were washed two times with PBS (Life Technologies) and 3 × 10^5^ cells were seeded at 4:1 ratio with previously frozen and freshly thawed allogeneic monocytes in a 96-well flat bottom plate (Corning). Next, BLZ945 (300 nM, Novartis) or Nivolumab (10 μg/mL, Bristol-Myers Squibb) was added alone or in combination to the wells. Matching concentrations of DMSO (Life Technologies) or a human IgG4 isotype control (Biolegend) were added as controls. In some experiments, different pharmacological inhibitors or blocking/neutralizing antibodies (Tables S1 and S2) or 10 μg/mL of human recombinant PD-L1 (AcroBiosystems) were tested in combination with Nivolumab. After 6 d, cells were harvested and proceeded to flow cytometric analysis.

In order to measure the intracellular production of M-CSF, freshly isolated PBMC were activated for 3 d with microbeads coated with anti-CD3/CD28 mAb (0.5–3 μL, Life Technologies) in a 96-well U bottom plate (Corning). During the final 12 h, GolgiPlug™ protein transport inhibitor (BD Biosciences) was added at 1:1500 dilution to the wells and cells were harvested for FACS analysis.

### Cytokine and chemokine analysis

In total, 43 human soluble factors were measured in the culture media harvested from three independent proliferation assays using a 37-parameter Bio-Plex™ Human Inflammation Panel including IL-27(p28), gp130/sIL-6Rβ, IL-34, IL-22, sIL-6Rα, IFN-α2, IFNγ, IL-26, MMP-2, IL-12(p40), IL-19, IL-20, IL-29(IFN-λ1), IL-35, IL-32, BAFF/TNFSF13B, IL-2, IL-11, APRIL/TNFSF13B, MMP-1, IFN-β, MMP-3, sCD163, Pentraxin-3, LIGHT/TNFSF14, TSLP, sCD30/TNFSF8, IL-8, IL-10, TWEAK/TNFSF12, Osteocalcin, IL-28A/(IFN-λ2), sTNF-R2, chitinase-3-like1, sTNF-R1, IL12(p70), and Osteopontin (Bio-Rad) and a 13-parameter LEGENDplex™ Human Anti-Virus Response Panel consisting of IL-1β, IL-6, TNF-α, IP-10 (CXCL10), IFN-λ1(IL-29), IL-8, IL12(p70), IFN-α2, IFN-λ2/3(IL28-A/B), GM-CSF, IFN-β, IL-10, and IFNγ (Biolegend), according to manufacturers' protocol. The overlapping factors between the two kits were adjusted. Levels of human soluble M-CSF were determined by ELISA (R&D systems). Concentrations of CXCL9 and CXCL10 in sera of treated or control mice were analyzed by a Legendplex assay (Biolegend).

### Treatment of human primary monocytes

To generate lymphocyte-conditioned media (Lym-med), 6.5 × 10^6^ monocyte-depleted human PBMC were co-cultured with allogeneic monocytes at 6:1 ratios in 3 mL culture medium per well in a six-well plate. The conditioned media were harvested and pooled after 6 d and stored in aliquots at −80°C. To evaluate the effects of Lym-med or M-CSF on primary human monocytes, 1 × 10^6^ freshly isolated monocytes were seeded in 600 μL culture medium in a 24-well plate (Corning) with various concentrations of Lym-med (5–20%) or recombinant human M-CSF (3.125–200 ng/mL, carrier-free, Biolegend), in the presence of DMSO or 1 μM BLZ945. Cells were harvested by carefully scraping the wells after 2 d (M-CSF) or 4 d (Lym-med) and phenotypic changes or intracellular protein levels of the monocytes were determined by flow cytometry.

### Flow cytometric analysis

Information regarding antibodies used for flow cytometry was summarized in Table S3. FACS stainings were performed in 96-well V bottom plates. Up to 1 × 10^6^ cells were placed in 96-well plates, washed two times with PBS, and stained for 20 min at room temperature with pre-mixed surface marker antibodies and life dead markers, near infra-red, Live/Dead fixable blue, or aqua dead cell marker (Invitrogen) in 20–50 μL PBS. After washing in PBS, cells were resuspended in FACS buffer (PBS+10% heat-inactivated FCS) and stored at 4°C before measurements. For intracellular stainings (i.c.), cells were fixed in BD Cytofix/Cytoperm buffer (BD Bioscience) for 30 min at room temperature and washed with 1× BD PermWash (BD Bioscience) buffer and incubated with intracellular antibodies in 1× BD PermWash buffer for 45 min at room temperature. After a final wash, cells were acquired at the BD LSRII cytometer (BD Bioscience) or the NovoCyte Flow Cytometer (ACEA Biosciences) and analyzed by FlowJo software (Tristar, Inc.) or NovoExpress software (ACEA), respectively.

### Multivariate analysis

All multivariate analyses were performed using the SIMCA software (version 14, Umetrics). In brief, raw data were modeled using PCA and the outliers were controlled based on the 95% confidence interval (CI) margins. The correlations between 44 human inflammatory soluble factors and frequencies of activated (CFSE^dim^CXCR3+) CD4^+^ and CD8^+^ T cells were demonstrated using loading plots of the PCA analysis. For the animal studies, frequencies of 59 splenic and intra-tumoral immune parameters were recorded first with FACS in control mice (*n* = 9), anti-PD-1-treated mice (*n* = 6), and mice that received combination treatments (*n* = 6), and differences between the chosen two groups were compared and summarized in the PCA analysis. Furthermore, immune profiles of control and different treatment groups were modeled and compared using the orthogonal partial least squares discriminant analysis (OPLS-DA). To investigate the importance of the immune subsets (X variables) to tumor weights (Y variables), we performed the OPLS analysis and selected the key contributors based on the variable importance scores (VIP), coefficient scores and cross-validation standard errors (cvSE).

### Patient datasets

In order to investigate the expression and prognostic values of the adenosine pathway, we analyzed publicly available expression datasets (‘R2: microarray analysis and visualization platform’, http://r2.amc.nl). In total, six independent patient cohorts were selected for correlation analyses (Spearman coefficients and two-tailed *t*-tests) and the largest patient cohort (Kocak, *n* = 649)^51^ was utilized to explore the prognostic values of *CD73* gene expression in neuroblastoma patients.

### Statistical analysis

Data were collected from several independent experiments and edited in GraphPad Prism, unless indicated differently. We first tested Normal distribution and equal variant of the datasets, followed by Student's *t*-tests or non-parametric, Mann–Whitney *U* tests (as stated in the figure legends). All results were presented as means±SD and representative histograms or pictures were selected based on the average values.

### Study approval

Mice were maintained in the Department of Comparative Medicine, Karolinska Institutet. All animal studies were approved by the local authorities and conducted under the ethical permit number N42/14.

### Data and materials availability

The data discussed in this publication have been deposited in NCBI's Gene Expression Omnibus^54^ (Edgar et al., 2002) and are accessible through GEO Series accession number GSE79485 (https://www.ncbi.nlm.nih.gov/geo/query/acc.cgi?acc=GSE79485).

CSF-1R inhibitor BLZ945 was provided through an MTA from Novartis.

## Supplementary Material

KONI_A_1232222_supplementary_data.zip
