# Supplementary material for: Regulation of myeloid cells by activated T cells determines the efficacy of PD-1 blockade
Source: Oncoimmunology. 2016 Sep 9;5(12):e1232222. doi: 10.1080/2162402X.2016.1232222 (PMC5214950; doi:10.1080/2162402X.2016.1232222)
Supplement: KONI_A_1232222_supplementary_data.zip [file koni-05-12-1232222-s001.zip › KONI_A_1232222_s02.docx]

**Supplementary materials**

**Supplemental Figure legends**

**Figure S1. (A)** The immune subsets in spleens and tumors of control or mice treated with anti-PD-1 therapy or combination therapy (BLZ945 + anti-PD-1) were modelled and plotted in PCA analysis in order to visualize outliers based on the 95% confidence intervals (CI). Frequencies of **(B)** CD4 T cells in spleens or tumors, **(C-D)** splenic suppressive myeloid cells or **(E-F)** intra-tumoral suppressive myeloid cells of wild type, control or treated mice were shown. *: *p*<0.05; **: *p*<0.01; ***: *p*<0.001; non-parametric Mann-Whitney U test. Each dot represented an individual mouse.

**Figure S2. (A)** Gene expression of *CXCL9, 10* and *11* in tumor tissues of TH-*MYCN* mice was correlated to expression of *CD11c*, and **(B)** gene expression of *CXCL9* in tumor tissues of was analyzed by microarray analysis and correlated with expression levels of *CD4*, *CD8A*, *CD19* or *B4galnt1* (GD-2 synthase). Correlation coefficients (R) were calculated by Spearman test and *p* values were calculated with two-tailed T tests. **(C)** Intra-tumoral frequencies of CD8 and CD4 T cells were shown in control mice or mice received combination therapy (BLZ945 + anti-PD-1) with or without blockade of CXCR3. *: *p*<0.05; non-parametric Mann-Whitney U test. Each dot represented an individual mouse.

**Figure S3. (A)** Freshly isolated PBMC were labeled with CFSE and activated with microbeads coated with agonistic anti-CD3/CD28 antibodies for 3 days. Activation of CD3+ T cells was measured by CFSE dilution and expression of CXCR3. **(B)** Representative plots were shown after Ficoll gradient centrifugation, with or without monocyte depleting agents. **(C)** Gating strategy of PBMC after 6 days of MLR activation and **(D)** expression levels of CSF-1R were compared between myeloid cells and lymphocytes. **(E)** Relative changes of CD4, CD8 and NK cell activations with IgG4, DMSO, Nivolumab (10 μg/ml) or BLZ945 (300 nM) were shown in 7 independent MLR experiments using monocyte-depleted PBMC. **Figure S4. (A)** Freshly isolated PBMC were labeled with CFSE and activated with increasing doses of agonistic anti-CD3/CD28-antibodies coated microbeads for 3 days and GolgiPlug inhibitor was added at 1:1500 for the final 12 hours. Intracellular expressions of Ki67 and M-CSF were measured by flow cytometry. **(B)** CFSE-pulsed human PBMC were activated in MLR with various numbers of allogeneic monocytes and activation of T cells was measured after 6 days by CFSE dilution and up-regulation of CXCR3. Concentrations of M-CSF in the matching experiments were determined by ELISA in technical duplicates.

**Figure S5. (A)** Freshly isolated human PBMC were activated in mixed lymphocyte reactions for 6 days, in the presence Nivolumab (10 μg/ml) in combination with various blocking antibodies or recombinant human PD-L1 (all at 10 μg/ml). Relative changes of CD8 T cell activation were compared to Nivolumab alone after 6 days. **(B)** Freshly purified human primary monocytes were cultured with recombinant human M-CSF or media conditioned by activated lymphocytes and expression of CD206 was compared after 48 hours between the DMSO group and cells treated with 1 μM BLZ945. **(C)** Expression of CD73 and CD39 were shown on lymphocytes or myeloid cells after MLR. **(D)** Human PBMC were activated in MLR with Nivolumab/BLZ945 combination (double), or in addition with CD73 inhibitor (APCP, 200 μM) or adenosine A2A receptor antagonist (SCH58621, 2.5 μM) triple combination and relative changes of CD8 T cell activation to Nivolumab alone were shown.

**Figure S6. (A)** Gene expression of *CD73* was correlated to *CD11b*, *CD4*, *CD8A*, *CD19* and tumor marker *Phox2b* genes, **(B)** gene expression of *M-CSF* was correlated to *PD-L1, CD73* and *CD39*, in tumor tissues of TH-*MYCN* mice and correlation coefficients were calculated by Spearman test and two-tailed T tests. **(C)** Survival of low-risk neuroblastoma patients based on *CD73* gene expression in tumor tissues was shown (Kocak dataset).

**Supplemental Tables**

Table S1. Pharmacological inhibitors utilized *in vitro*.

|  | **Pharmacological Inhibitors** | | | | | | | |
| --- | --- | --- | --- | --- | --- | --- | --- | --- |
| Names | **APCP** ^1^ | **SCH58621** ^1^ | **1-DL-MT** ^1^ | **Catalase** ^1^ | **SOD** ^1^ | **nor-NOHA** ^2^ | **1400W** ^1^ | **Celecoxib** ^3^ |
| Targets | CD73 | AdeR A2A ^I^ | IDO ^II^ | H_2_O_2_ | Superoxide | Arginase-1 | iNOS ^III^ | COX-2 ^IV^ |
| Doses | 200 µM | 2.5 µM | 100 µM | 200 IU/ml | 200 IU/ml | 200 µM | 200 µM | 5 µM |
| Manufacturers: ^1^ Sigma-Aldrich; ^2^ Calbiochem; ^3^ Cayman Chemicals.  Notes: ^I^ Adenosine Receptor A2A; ^II^ Indoleamine-2,3-dioxygenase; ^III^ Inducible nitric oxide; ^IV^ Cyclooxygenase-2. | | | | | | | | |

Table S2. Blocking or neutralizing antibodies utilized *in vitro*.

|  | **Blocking/neutralizing antibodies** | | | | | |
| --- | --- | --- | --- | --- | --- | --- |
| Names | **αCTLA-4**  **(Ipilimumab)** ^1^ | **αPGE2** ^2^ | **αDC-SIGN** ^3^ | **αTGF-β** ^3^ | **αIL-10** ^4^ | **αCD80** ^4^ |
| Names | **αCD137** ^4^ | **αMIC A/B** ^4^ | **αCXCR3** ^4^ | **αNKp30** ^4^ | **αNKp44** ^4^ | **αNKp46** ^4^ |
| Concentrations | 10 µg/ml | | | | | |
| Manufacturers: ^1^ Bristol-Myer Squibb; ^2^ Cayman Chemicals; ^3^ R&D Systems; ^4^ Biolegend. | | | | | | |

**Table S3.** Antibodies and dyes used for flow cytometry.

| **Antigen** | **Clone** | **Isotype** | **Fluorochrome** |
| --- | --- | --- | --- |
| *anti-human*  CD3 ^1^ | UCHT1 | Mouse IgG1, κ | Pacific Blue |
| CD4 ^1^ | A161A1 | Rat IgG2b, κ | PerCp-Cy5.5 |
| CD11b ^2^ | ICRF44 | Mouse IgG1, κ | APC-Cy7 |
| CD16 ^3^ | 3C8 | Mouse IgG1 | APC |
| CD39 ^1^ | A1 | Mouse IgG1, κ | PE-Cy7 |
| CD56 ^1^ | HCD56 | Mouse IgG1, κ | PE-Cy7 |
| CD73 ^1^ | AD2 | Mouse IgG1, κ | Pacific Blue |
| CD80 ^1^ | 2DF10 | Mouse IgG1, κ | Brilliant Violet 421 |
| CD115 ^1^ (CSF-1R) | 9-4D2-1E4 | Rat IgG1, κ | PE |
| CD137L ^1^ | 5F4 | Mouse IgG1, κ | PE |
| CD183 ^1^ (CXCR3) | G025H7 | Mouse IgG1, κ | Pacific Blue |
| CD206 ^1^ | 15-2 | Mouse IgG1, κ | PerCp-Cy5.5 |
| CD274 ^2^ (PD-L1) | MIH1 | Mouse IgG1, κ | PE-Cy7 |
| DC-SIGN ^1^ | 9E9A8 | Mouse IgG2a, κ | PerCp-Cy5.5 |
| HLA-DR ^3^ | MEM-12 | Mouse IgG1, κ | FITC |
| IDO ^4^ | 700838 | Mouse IgG1 | Alexa Fluor 488 |
| IFN-γ ^1^ | 4S.B3 | Mouse IgG1, κ | PE-Cy7 |
| M-CSF ^4^ | 26786 | Mouse IgG2a | APC |
| Ki67 ^1^ | Ki-67 | Mouse IgG1, κ | Brilliant Violet 421 |
| *anti-mouse*  CD3e ^1^  CD4 ^1^  CD8 ^2^ | 145-2C11  GK1.5  53-6.7 | Arm. Hamster IgG  Rat IgG2b, κ  Rat IgG2b, κ | Brilliant Violet 421  PerCp-Cy5.5  APC-Cy7 |
| CD11b ^1^ | M1/70 | Rat IgG2b, κ | APC |
| CD19 ^1^ | 6D5 | Rat IgG2a, κ | AF700 |
| CD25 ^1^ | 3C7 | Rat IgG2b, κ | FITC |
| CD45 ^1^  CD45R/B220 ^1^ | 30-F11  RA3-6B2 | Rat IgG2b, κ  Rat IgG2a, κ | APC, PerCP, PO  PerCP-Cy5.5 |
| CD49b ^1^ | HMa2 | Rat IgG2b, κ | PE |
| CD69 ^1^ | H1.2F3 | Arm. hamster IgG, λ3 | PE-Cy7 |
| CD86 ^1^ | PO3 | Rat IgG2b, κ | FITC |
| CD115 (CSF-1R) ^5^ | AFS98 | Rat IgG2b, κ | PE-eFluor610 |
| CD183 (CXCR3) ^1^ | CXCR3-173 | arm. Hamster IgG | Brilliant Violet 510 |
| CD206 ^1^ | C068C2 | Rat IgG2b, κ | FITC |
| CXCL9 ^1^ | MIG-2F5.5 | arm. hamster IgG, κ | PE |
| F4-80 ^1^ | BM8 | Rat IgG2a, κ | PE |
| I-A/I-E ^1^ | M5/14.1.5.2 | Rat IgG2b, κ | APC-Cy7 |
| Ly6c ^2^ | AL-21 | Rat IgM, κ | PerCP-Cy5.5 |
| Ly6g ^2^ | 1A8 | Rat IgG2a, κ | PE-Cy7 |
| PD-L1 ^1^ | 10F.9G2 | Rat IgG2b, κ | PE-Cy7 |
| PD-1 ^1^ | RMP1-30 | Rat IgG2b, κ | PE |
|  |  |  |  |
| Live/Dead markers ^6^ |  |  | Near Infra-red, Aqua, or Live/Dead fixable blue |
| Manufacturers: ^1^ Biolegend; ^2^ BD Biosciences;  ^3^ ImmunoTools; ^4^ R&D Systems; ^5^ eBioscience; ^6^Invitrogen | | | |
|  | | | |

| **Table S4.** 59 immune subsets monitored in TH-*MYCN* mice. | | | | |
| --- | --- | --- | --- | --- |
| Organ | T cells | | Myeloid cells | |
| Spleen | CD4 total  CD4+CD25+  CD4+CD69+ | CD8 total  CD8+CD25+  CD8+CD69+ | % in CD11b+ | % in F4/80 |
|  |  |  | Ly6G+  Ly6C+  MHCII (I-A/I-E)+  F4/80+  PD-L1+  PD-L1high  PD-L1medium  CD86+  PD-L1+CSF-1R+ | CD206+  CSF1R+  PD-L1+  PD-L1high  PD-L1medium  MHCII (I-A/I-E)+  CD86+  PD-L1+CSF-1R+ |
|  |  |  | % in CD45+ | Others |
|  |  |  | CD11b+Ly6G+  CD11b+Ly6C+  CD11b+F4/80+  CD11b+ total | CSF-1R+ in Ly6G+  CSF-1R+ in Ly6C+ |
| Tumors | CD4 total  CD4+CD25+  CD4+PD1+  CD4/CD8 ratio | CD8 total,  CD8+CD69+  CD8+PD1+ | Same as above | |
