# Supplementary material for: Regulation of myeloid cells by activated T cells determines the efficacy of PD-1 blockade
Source: Oncoimmunology. 2016 Sep 9;5(12):e1232222. doi: 10.1080/2162402X.2016.1232222 (PMC5214950; doi:10.1080/2162402X.2016.1232222)
Supplement: KONI_A_1232222_supplementary_data.zip [file koni-05-12-1232222-s001.zip › KONI_A_1232222_s03.pptx]

## Slide 1
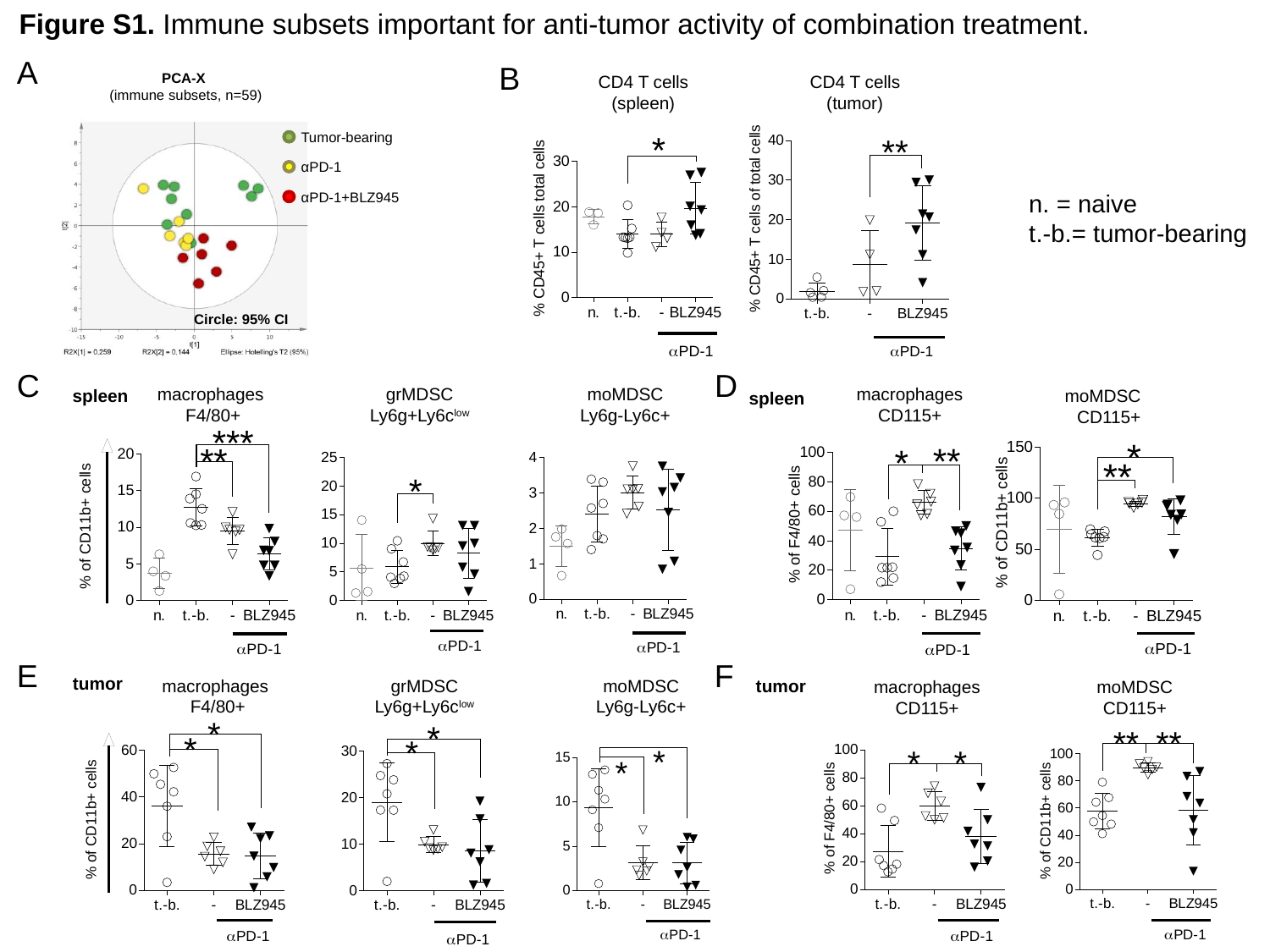

Figure S1. Immune subsets important for anti-tumor activity of combination treatment.
A
B
PCA-X
(immune subsets, n=59)
Tumor-bearing
αPD-1
αPD-1+BLZ945
Circle: 95% CI
CD4 T cells
(spleen)
CD4 T cells
(tumor)
n. = naive
t.-b.= tumor-bearing
C
D
macrophages
F4/80+
grMDSC
Ly6g+Ly6clow
moMDSC
Ly6g-Ly6c+
spleen
macrophages
CD115+
moMDSC
CD115+
spleen
E
F
tumor
macrophages
F4/80+
grMDSC
Ly6g+Ly6clow
moMDSC
Ly6g-Ly6c+
tumor
macrophages
CD115+
moMDSC
CD115+

## Slide 2
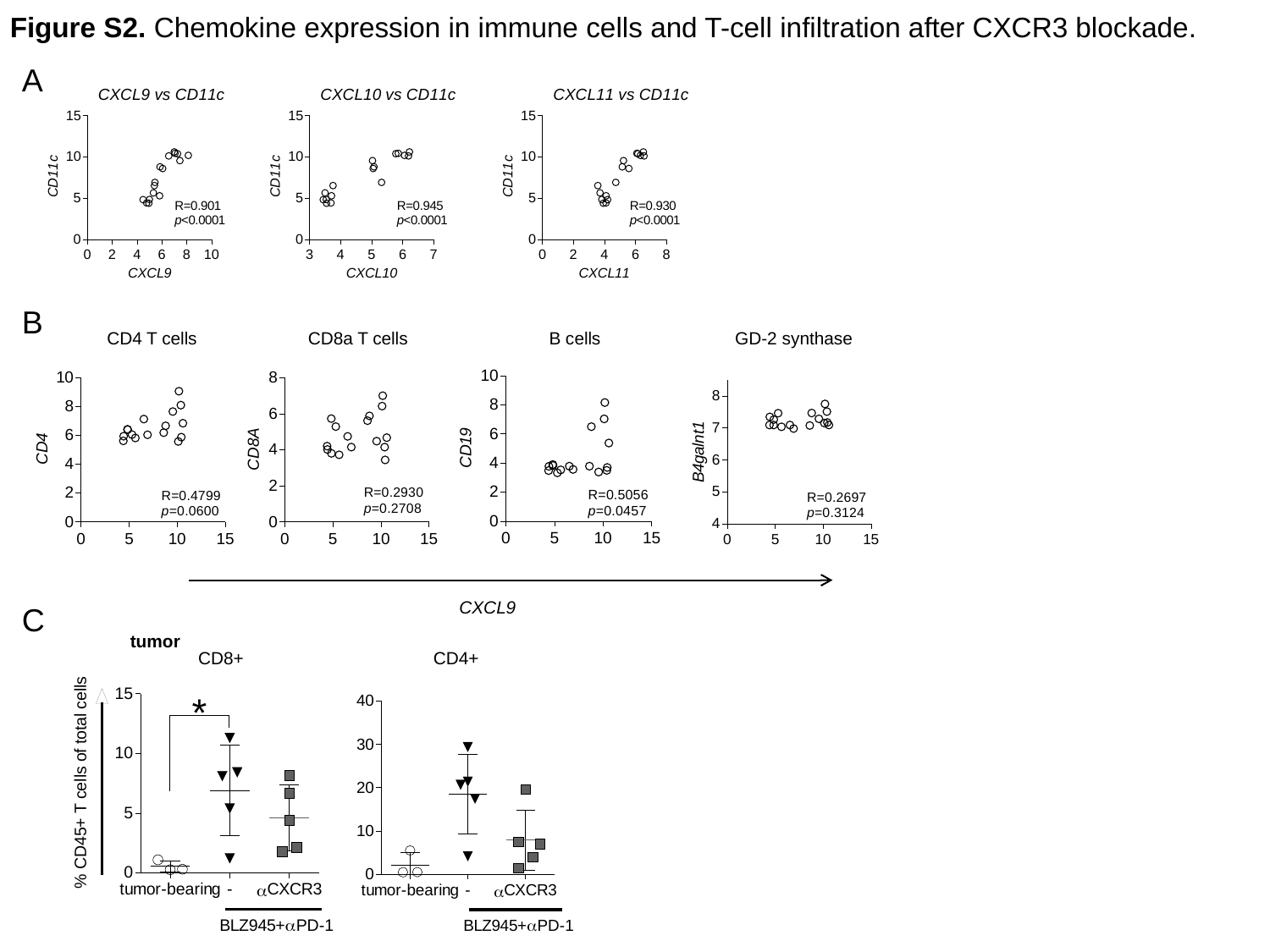

Figure S2. Chemokine expression in immune cells and T-cell infiltration after CXCR3 blockade.
A
B
CD4 T cells
CD8a T cells
B cells
GD-2 synthase
CXCL9
C
tumor
CD8+
CD4+

## Slide 3
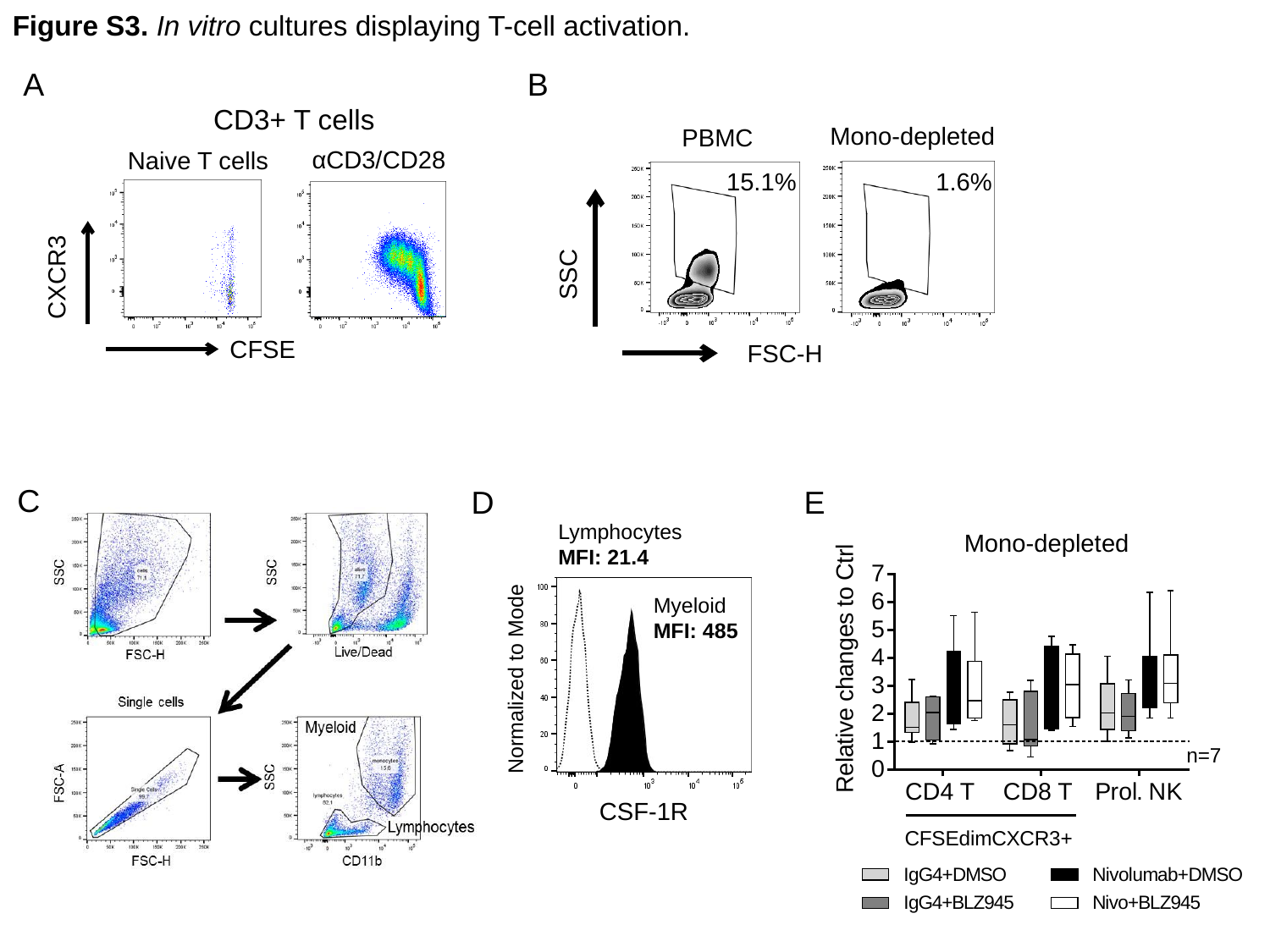

Figure S3. In vitro cultures displaying T-cell activation.
A
B
CD3+ T cells
CXCR3
CFSE
αCD3/CD28
Naive T cells
Mono-depleted
1.6%
PBMC
15.1%
SSC
FSC-H
C
D
E
Lymphocytes
MFI: 21.4
Normalized to Mode
Myeloid
MFI: 485
CSF-1R
Mono-depleted

## Slide 4
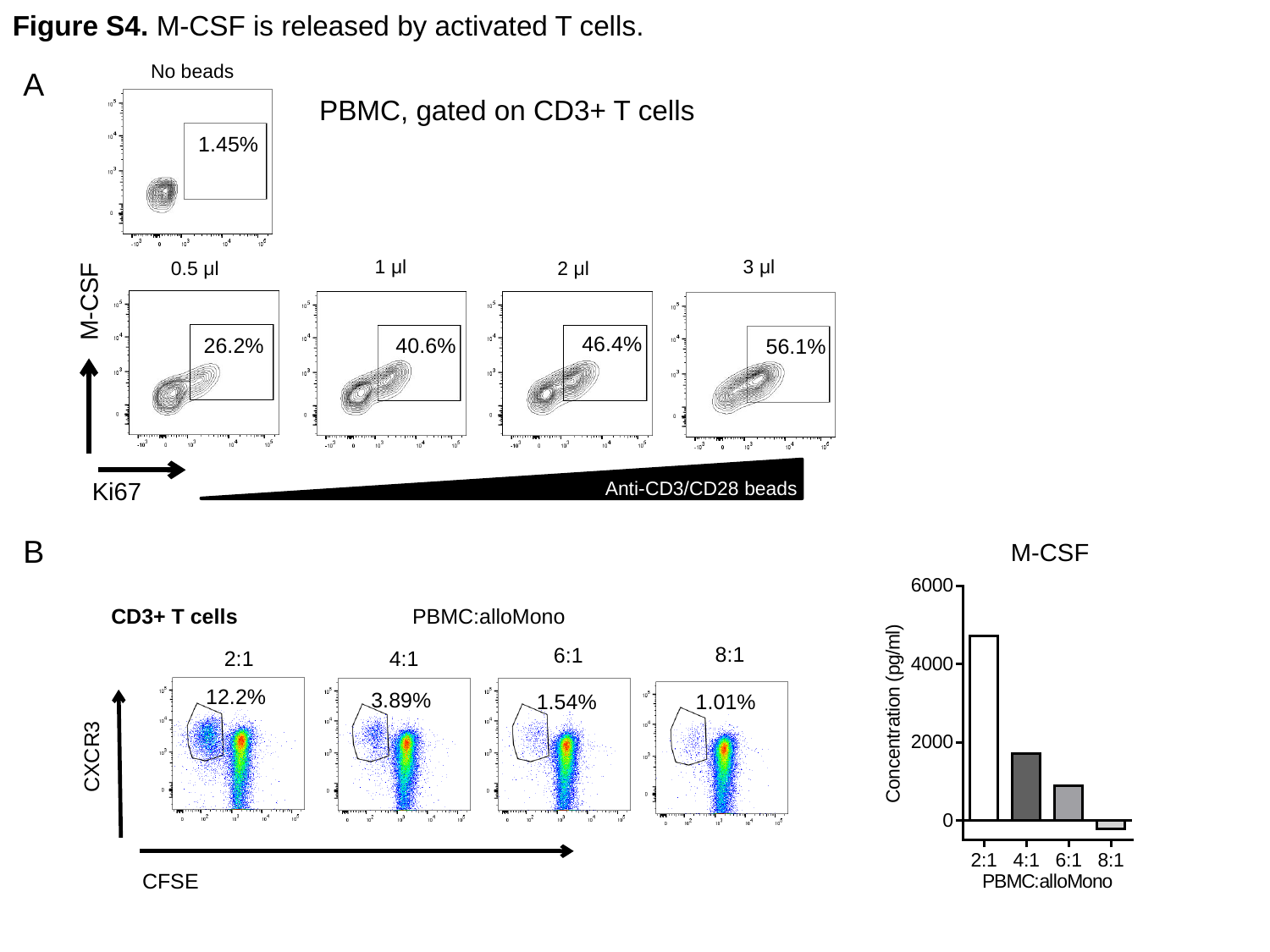

Figure S4. M-CSF is released by activated T cells.
No beads
1.45%
PBMC, gated on CD3+ T cells
M-CSF
Ki67
1 μl
40.6%
3 μl
56.1%
0.5 μl
26.2%
2 μl
46.4%
Anti-CD3/CD28 beads
A
B
M-CSF
CD3+ T cells
PBMC:alloMono
8:1
1.01%
6:1
1.54%
2:1
12.2%
4:1
3.89%
CXCR3
CFSE

## Slide 5
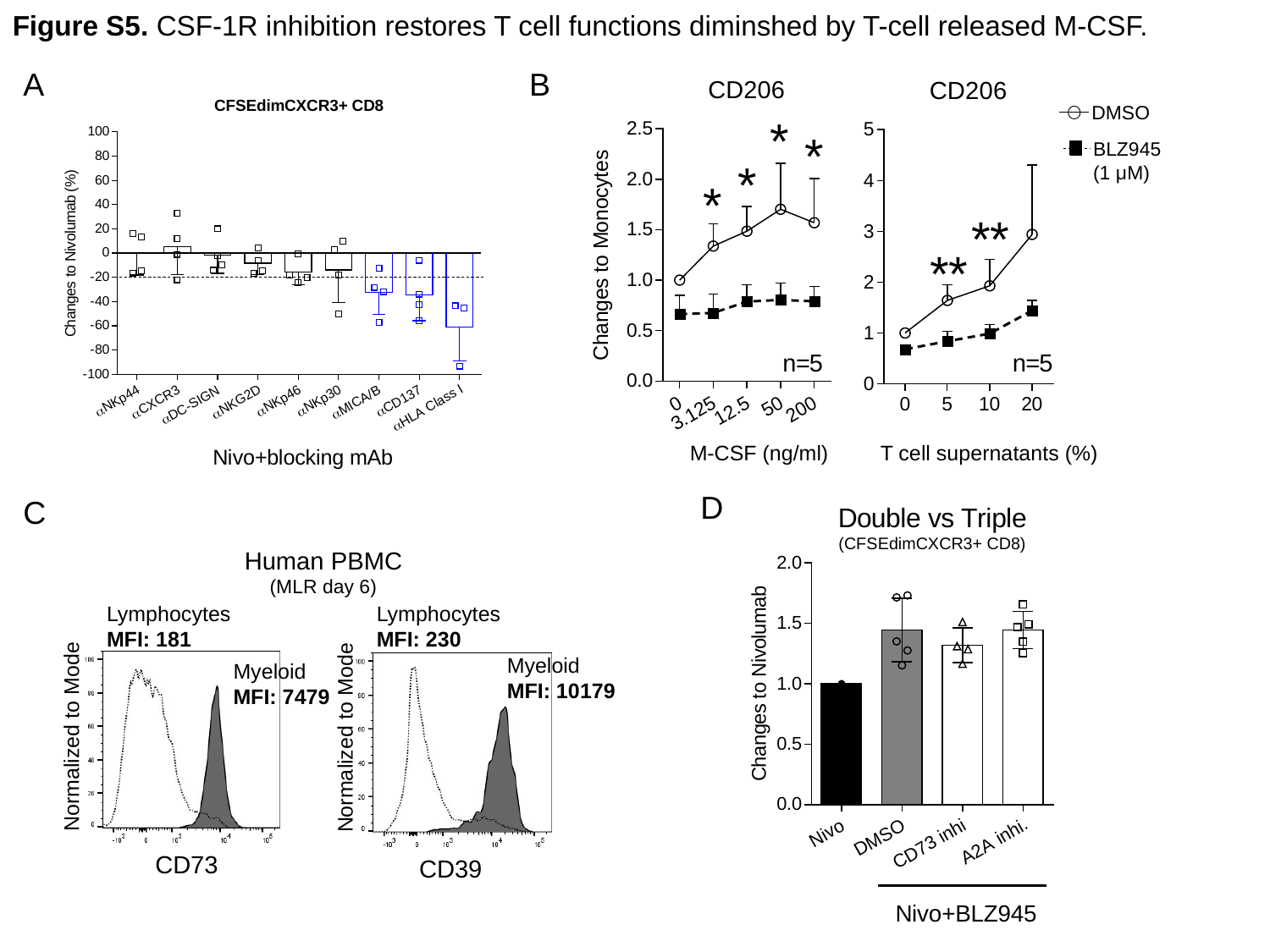

Figure S5. CSF-1R inhibition restores T cell functions diminshed by T-cell released M-CSF.
A
B
M-CSF (ng/ml)
T cell supernatants (%)
DMSO
BLZ945 (1 μM)
D
C
Human PBMC
(MLR day 6)
Lymphocytes
MFI: 181
Myeloid
MFI: 7479
Normalized to Mode
CD73
Lymphocytes
MFI: 230
Myeloid
MFI: 10179
Normalized to Mode
CD39

## Slide 6
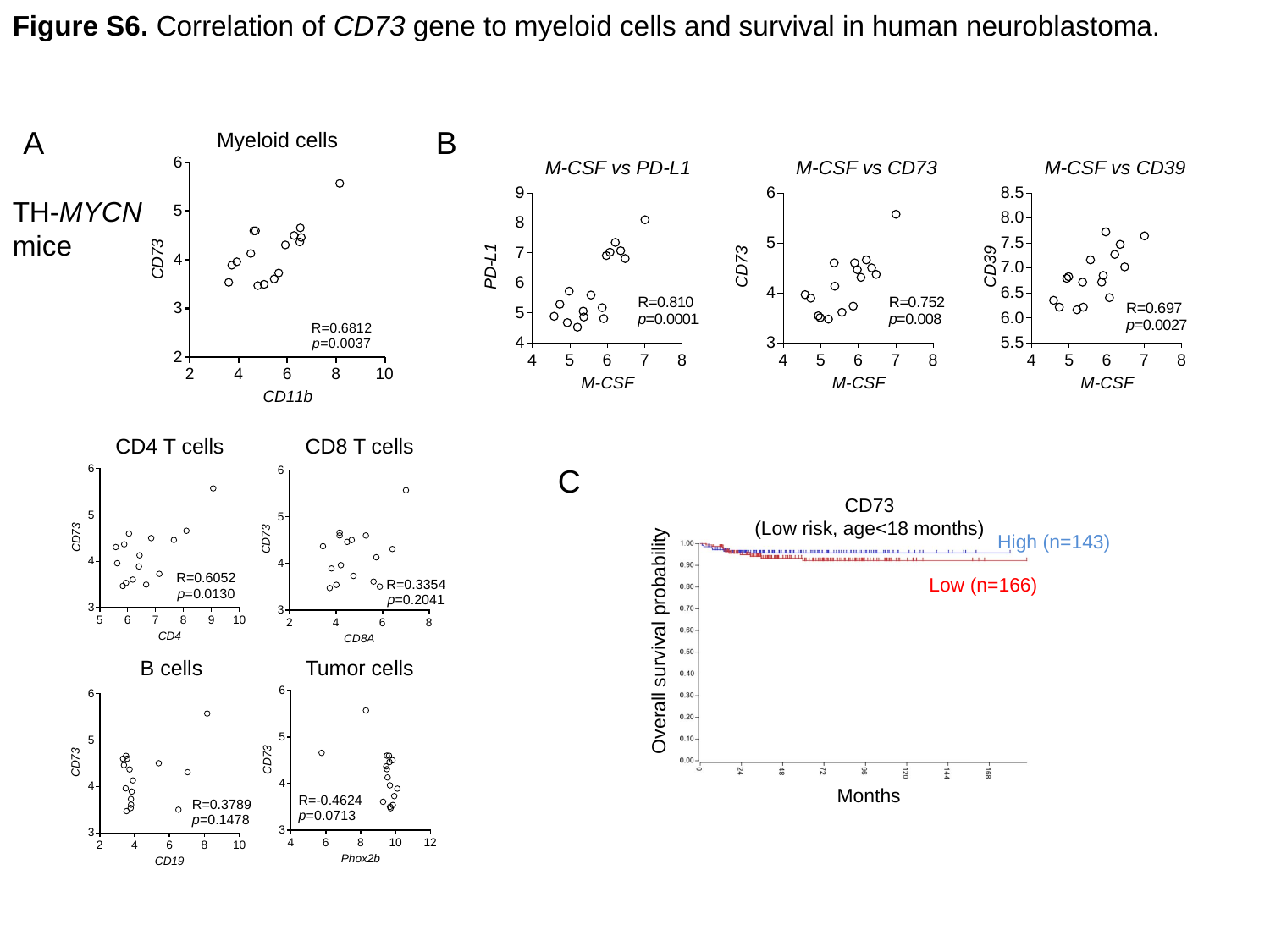

Figure S6. Correlation of CD73 gene to myeloid cells and survival in human neuroblastoma.
A
B
Myeloid cells
TH-MYCN
mice
CD8 T cells
CD4 T cells
C
B cells
Tumor cells
